# The REinfection in COVID‐19 Estimation of Risk (RECOVER) study: Reinfection and serology dynamics in a cohort of Canadian healthcare workers

**DOI:** 10.1111/irv.12997

**Published:** 2022-05-05

**Authors:** Étienne Racine, Guy Boivin, Yves Longtin, Deirdre McCormack, Hélène Decaluwe, Patrice Savard, Matthew P. Cheng, Marie‐Ève Hamelin, Julie Carbonneau, Fazia Tadount, Kelsey Adams, Benoîte Bourdin, Sabryna Nantel, Vladimir Gilca, Jacques Corbeil, Gaston De Serres, Caroline Quach‐Thanh

**Affiliations:** ^1^ Department of Epidemiology, Biostatistics and Occupational Health McGill University Montreal Quebec Canada; ^2^ Sainte‐Justine Hospital Health and Research Center Montreal Quebec Canada; ^3^ Department of Microbiology‐Immunology and Infectious Diseases Laval University Quebec City Quebec Canada; ^4^ Infectious and Immune Diseases Axis Research Center of the Centre Hospitalier de l'Université Laval Quebec City Quebec Canada; ^5^ Jewish General Hospital and Lady Davis Research Institute Montreal Quebec Canada; ^6^ McGill University Health Center Montreal Quebec Canada; ^7^ Immune Diseases and Cancer Axis Sainte‐Justine Hospital University Health and Research Center Montreal Quebec Canada; ^8^ Department of Pediatrics University of Montreal Montreal Quebec Canada; ^9^ Department of Microbiology, Infectious Diseases and Immunology University of Montreal Montreal Quebec Canada; ^10^ Immunopathology Axis Research Center of the Centre Hospitalier de l'Université de Montréal Montreal Quebec Canada; ^11^ Infectious Disease Service Centre Hospitalier de l'Université de Montréal Montreal Quebec Canada; ^12^ Divisions of Infectious Diseases and Medical Microbiology, McGill University Health Center McGill University Montreal Quebec Canada; ^13^ McGill Interdisciplinary Initiative in Infection and Immunity Montreal Quebec Canada; ^14^ Department of Microbiology, Infectiology and Immunology University of Montreal Montreal Quebec Canada; ^15^ Quebec National Public Health Institute Quebec City Quebec Canada; ^16^ Department of Molecular Medicine, Big Data Research Center, Institute Intelligence and Data Laval University Quebec City QC Canada; ^17^ Infectiology Research Center of the Centre Hospitalier Universitaire de Québec Quebec City QC Canada; ^18^ Department of Social and Preventive Medicine Laval University Quebec City Quebec Canada; ^19^ Department of Microbiology, Infectious Diseases, and Immunology University of Montreal Montreal Quebec Canada; ^20^ Sainte‐Justine Hospital University Health and Research Center Montreal Quebec Canada

**Keywords:** COVID‐19, prospective studies, reinfection, SARS‐CoV‐2, serology

## Abstract

**Background:**

Understanding the immune response to natural infection by SARS‐CoV‐2 is key to pandemic management, especially in the current context of emerging variants. Uncertainty remains regarding the efficacy and duration of natural immunity against reinfection.

**Methods:**

We conducted an observational prospective cohort study in Canadian healthcare workers (HCWs) with a history of PCR‐confirmed SARS‐CoV‐2 infection to (i) measure the average incidence rate of reinfection and (ii) describe the serological immune response to the primary infection.

**Results:**

Our cohort comprised 569 HCWs; median duration of individual follow‐up was 371 days. We detected six cases of reinfection in absence of vaccination between August 21, 2020, and March 1, 2022, for a reinfection incidence rate of 4.0 per 100 person‐years. Median duration of seropositivity was 415 days in symptomatics at primary infection compared with 213 days in asymptomatics (*p* < 0.0001). Other characteristics associated with prolonged seropositivity for IgG against the spike protein included age over 55 years, obesity, and non‐Caucasian ethnicity.

**Conclusions:**

Among unvaccinated healthcare workers, reinfection with SARS‐CoV‐2 following a primary infection remained rare.

## Abstract 2

SARS‐CoV‐2 reinfections remained rare events among unvaccinated healthcare workers. Prior natural infection confers some protection against reinfection and clinical disease, but waning of serum antibodies suggests this protection may not last in the long term.

## 1 INTRODUCTION

Since its appearance in Wuhan (China) in December 2019, the severe acute respiratory syndrome coronavirus 2 (SARS‐CoV‐2) that causes coronavirus disease (COVID‐19) spread into a global pandemic, leading to more than 480 million reported cases and over 6.1 million confirmed deaths as of March 29, 2022, according to the WHO.^1^ COVID‐19 continues to exert a high burden on healthcare systems across the world because of effective interhuman transmissibility and clinical illness that leads to hospitalization in severe cases. To curb virus transmission and avoid overwhelming healthcare systems, different non‐pharmacological interventions (NPIs) have been implemented, including physical/social distancing, improved hand hygiene adherence, mask mandates, business and school closures, citywide lockdowns, and international border closures. Although these mitigation measures have caused significant economic, social, and health‐related adverse effects,^2^ they remain necessary until a sufficient proportion of individuals become protected against severe COVID‐19. With the recent emergence of the highly transmissible Omicron variant, this proportion could be higher than 90%.^3^

Protection against SARS‐CoV‐2 infection may be acquired by recovering from a previous episode of natural infection. However, the duration of natural immunity is still uncertain, and infection of a large proportion of the population may not suffice to achieve collective immunity, particularly when facing emerging variants. This is of concern because of unequal vaccine distribution across the world^4^; significant levels of vaccine hesitancy, notably in Europe and the United States^5^; and the potential ability of recent variants to escape immunity specific to older variants. Therefore, it is important to determine if individuals with a history of PCR‐confirmed SARS‐CoV‐2 infection are protected against reinfection and viral shedding and if so, how long this protection lasts.

Several large‐scale prospective and retrospective cohort studies have recently addressed SARS‐CoV‐2 reinfection epidemiology in both healthcare workers (HCWs)^6^, ^7^, ^8^, ^9^ and general populations.^10^, ^11^, ^12^, ^13^, ^14^, ^15^ All these studies report that reinfections are generally uncommon events (less than 1% risk over several months following primary infection) and that a history of previous infection confers protection against future infection (ranging from 82% to 93%). This protection persists for at least a few months, but its long‐term duration remains largely unknown. Recent evidence suggests that the risk of reinfection could be significantly higher with the new Omicron variant compared with previous variants.^16^

The primary objective of the REinfection in COVID‐19 Estimation of Risk (RECOVER) study is to estimate the incidence rate of reinfection with SARS‐CoV‐2 in a population of HCWs with a history of PCR‐confirmed SARS‐CoV‐2 infection, acquired during the first or second wave of the pandemic. We describe in detail all cases of reinfection detected during the first 18 months of the study, estimate the reinfection incidence rate in unvaccinated HCWs, and describe the serological response following primary infection.

## 2 METHODS

### 2.1 Study population

The RECOVER study is an observational prospective cohort study of HCWs with a history of PCR‐confirmed SARS‐CoV‐2 infection. Eligible HCWs comprised any professional working in the Greater Montreal (Quebec, Canada) area healthcare facilities. These included physicians, nurses, patient attendants, therapists, technicians, maintenance employees, food workers, administrative personnel, and researchers.

HCWs were recruited between August 17, 2020, and April 8, 2021, primarily through the McGill University Health Center (MUHC)/Centre hospitalier universitaire Sainte‐Justine (CHUSJ) Vaccine Center. Additional recruitment took place at the Centre hospitalier de l'Université de Montréal (CHUM) and the Jewish General Hospital (JGH).

**FIGURE 1:**
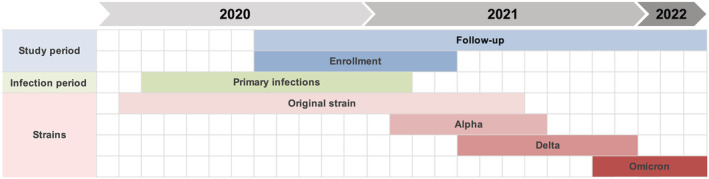
Timeline of the RECOVER study, overlaid with variants in circulation during the study period. Enrollment of participants was from August 17, 2020, to April 8, 2021. Follow‐up period was from August 17, 2020, to March 1, 2022. Enrolled participants acquired their primary infection between March 6, 2020, and February 14, 2021. Approximate periods for circulation of variants were derived from provincial surveillance data

Prospective participants were excluded if (i) they were no longer working in a healthcare setting or had been furloughed as a preventive measure at enrollment, (ii) they were not fluent in either French or English, (iii) they had no access to a cell phone or Internet, (iv) they were participating in a clinical trial for preventive treatment for COVID‐19, and/or (v) they received a COVID‐19 vaccine prior to enrollment. Planned follow‐up period was 12 months for all participants and 18 months for participants that remained unvaccinated. The study timeline is illustrated in Figure 1.

### 2.2 Data collection, management, and analysis

Baseline demographic, clinical, and biological data were obtained from each participant at enrollment (D0). Blood samples were drawn at D0 for assessment of immune response following primary infection. Every 2 weeks, an electronic questionnaire was sent to participants inquiring about new COVID‐19 symptoms, who need to consult a physician because of symptoms and history of recent significant exposure to a confirmed case of COVID‐19. Significant exposure was defined as at least 15 min within 2 m of a confirmed infectious case without proper use of recommended personal protective equipment (PPE).

Quarterly in‐person visits were planned at D90, D180, D270, and D360, where participants provided the following updates, if any: change in workplace or work duties, change in residual symptoms, new influenza‐like illness, new medical condition, new medication, new vitamin intake, and new vaccination. Blood samples for antibody serology and immune response assessment were drawn.

Participants were asked to contact the research team between planned follow‐ups if any of the following events occurred: (i) new symptoms onset, (ii) close contact with a confirmed case of COVID‐19, (iii) significant exposure to a COVID‐19 patient in the workplace without proper use of PPE, or (iv) vaccination with any COVID‐19 vaccine. If a participant reported new symptoms, a nasopharyngeal (NP) swab was performed. If the result was positive, an acute visit (2–4 days post symptoms onset) and convalescent visit (28–42 days post symptoms onset) were scheduled to obtain blood samples and information related to a possible reinfection. For the first 30 participants* who reported a significant exposure, a follow‐up visit was scheduled 4–7 days post exposure to collect an NP swab to ascertain asymptomatic reinfection.

Anonymized data were collected, stored, and managed using REDCap.^17^ Statistical analyses were performed with R Version 4.1.2 and RStudio Version 2021.09.1. Kaplan–Meier survival analysis, Kaplan–Meier curves, and Cox regression models were produced with the *survival* and *ggplot2* R packages.

### 2.3 Outcomes

Primary outcomes were possible, probable, or confirmed reinfection with SARS‐CoV‐2, in absence of vaccination. Possible reinfection was defined as a positive PCR test less than 90 days after first positive PCR. Probable reinfection was defined as a positive PCR test 90 days or more after the first positive PCR. Confirmed reinfection required either (i) evidence of infection by a known distinct variant or (ii) evidence of infection by a variant that was not circulating at time of first infection or (iii) confirmation that primary infection and reinfection strains were different by whole genome sequencing. Participants that received any vaccination against SARS‐CoV‐2 during follow‐up were right‐censored for reinfection outcomes at reception of the first dose. Our secondary outcome was serology status for IgG against the receptor‐binding domain (RBD) of the SARS‐CoV‐2 spike as a function of time since primary infection, in participants that remained unvaccinated; in Quebec, there was no mandatory vaccination for HCWs to continue working.

### 2.4 Laboratory methods

Qualitative reverse‐transcriptase PCR (RT‐PCR) was performed on all samples. Upon reinfection, both the original and reinfecting strains were sequenced, whenever possible, for phylogenetic studies to determine if they differed. For RT‐PCR analysis, primary infection strains were obtained from the Laboratoire de santé publique du Québec (LSPQ) or from the laboratory where original testing was performed when unavailable from the LSPQ.

Antibody detection was performed on blood samples taken at D0 and at each quarterly visit. For symptomatic reinfections, acute and convalescent sera were collected for antibody testing. IgG levels were detected using an in‐house, validated ELISA test based on the RBD of the spike protein. Validation of our in‐house ELISA was performed using a panel of 81 serum samples provided by the National Microbiology Laboratory of Canada; our RBD assay had a sensitivity of 95% and specificity of 100%. Participants were considered seropositive if the optical density (OD) was higher than the mean OD of negative controls plus three standard deviations. Controls were negative sera obtained in the pre‐pandemic era.

## 3 PATIENT CONSENT STATEMENT

Written consent was obtained from all participants at enrollment and was further reviewed and confirmed at each quarterly visit. This study was approved by the ethics committee of the Sainte‐Justine Hospital Research Center under the Nagano platform project number MP‐21‐2021‐3035.

## 4 RESULTS

### 4.1 Demographics and clinical data

Our cohort comprised 569 HCWs. The median duration of individual follow‐up was 371 days (IQR: 363–378 days). The demographic characteristics of our cohort are presented in Table 1. Participants were in majority female and Caucasian, and median age was 42 years. Most participants worked in acute‐care hospitals or in public long‐term care facilities. The most reported professions were nurse/paramedic, patient care attendant, and physician/medical resident.

**TABLE 1:** Demographic characteristics of the RECOVER cohort

Sex at birth	*n* (%)
Female	472 (83.0)
Male	97 (17.0)
Age	Years
Median (IQR)	42 (18)
Range	18–75
Ethnicity	n (%)
Caucasian	451 (79.3)
Middle‐Eastern	14 (2.5)
Latino	22 (3.9)
Asian	38 (6.7)
Black/African‐American	35 (6.2)
First Nations	1 (0.2)
Other	8 (1.4)
Workplace	*n* (%)
Hospital	307 (54.0)
Public long‐term care facility	139 (24.4)
Community health center	50 (8.8)
Private care facility	12 (2.1)
Other	61 (10.7)
Staff group	*n* (%)
Medical doctor/resident	67 (11.8)
Nurse/paramedic	229 (40.2)
Patient care attendant	73 (12.8)
Therapist/other healthcare professional in regular close contact with patients^a^	80 (14.1)
Education/recreation	11 (1.9)
Pharmacist/pharma assistant	9 (1.6)
Technician	20 (3.5)
Research staff	7 (1.2)
Administration/management	26 (4.6)
Maintenance/housekeeping	16 (2.8)
Food service	5 (0.9)
Other	26 (4.6)

*Note*: The total number of enrolled HCWs is 569.

^a^ Includes physiotherapists, occupational therapists, respiratory therapists, kinesiologists, nutritionists, social workers, psychologists, speech therapists, audiologists, and electrophysiologists.

Medical/lifestyle data of our cohort are presented in Table 2. Eighty‐two participants reported at least one medical condition considered a risk factor for severe COVID‐19 illness.^18^ Most participants were either overweight or obese and did not smoke tobacco products (including vaping products), cannabis, or other drugs. Nearly a third of participants reported regular vitamin D intake.

**TABLE 2:** Risk factors for severe COVID‐19 illness and lifestyle data

Medical conditions associated with increased risk of severe COVID‐19 illness^a^	*n* (%)
Hypertension	50 (8.8)
Chronic heart disease	3 (0.5)
Diabetes	21 (3.7)
Chronic lung disease^b^	8 (1.4)
Chronic kidney disease	1 (0.2)
Chronic liver disease	7 (1.2)
Immune system suppression	7 (1.2)
Other^c^	10 (1.8)
At least 1 comorbidity	82 (14.4)
Body mass index	*n* (%)
Underweight (BMI < 18.5 kg/m^2^)	8 (1.4)
Normal weight (18 ≤ BMI < 25 kg/m^2^)	246 (43.2)
Overweight (25 ≤ BMI < 30 kg/m^2^)	174 (30.6)
Obese (BMI ≥ 30 kg/m^2^)	141 (24.8)
Lifestyle	*n* (%) or UI
Smoking/vaping^d^	43 (7.6)
Vitamin D regular intake	182 (32.0)
Median weekly dose (IQR)	7000 UI (3000)

^a^ There were no active cancers in our cohort, so this category is not included.

^b^ Excluding asthma.

^c^ Includes pregnancy, thalassemia and sickle cell anemia.

^d^ Includes cannabis/cannabis products.

### 4.2 Primary COVID‐19 illness

Data regarding primary infections are reported in Table 3. In most participants, the primary infection resulted in symptomatic COVID‐19 illness. The median duration of acute symptoms was 14 days. Exposures leading to the primary infection occurred mostly in the workplace or in the household.^†^ Thirty‐four participants required hospitalization to manage their primary illness. Obesity was the only significant individual risk factor for hospitalization identified through multivariate logistic regression [adjusted OR = 2.80 (1.15–7.02)] (see Supporting Information for details). Median time between primary infection and enrollment was 177 days. Seventy‐four participants were enrolled less than 90 days after primary infection.

**TABLE 3:** Description of the initial SARS‐CoV‐2 infections/COVID‐19 illness episodes

Symptomology	*n* (%)
At least 1 symptom	541 (95.1)
Paucisymptomatic (1–7 symptoms)	225 (39.5)
Polysymptomatic (8 symptoms or more)	316 (55.5)
Asymptomatic	28 (4.9)
Reported symptoms	*n* (%)^a^
Fever	288 (53.2)
Fatigue	482 (89.1)
Myalgia	365 (67.5)
Cough	352 (65.1)
Sore throat	260 (48.1)
Dyspnea	241 (44.5)
Nasal congestion	217 (40.1)
Anosmia	394 (72.8)
Ageusia	340 (62.8)
Chest pain	183 (33.8)
Headache	414 (76.5)
Dizziness	173 (32.0)
Diarrhea	190 (35.1)
Nausea	146 (27.0)
Vomiting	51 (9.4)
Abdominal pain	92 (17.0)
Loss of appetite	261 (48.2)
Duration of acute symptoms, if present^‡^	Days
Median	14
25th–75th percentiles	7–21
Location of exposure	*n* (%)
Occupational	425 (74.7)
Household	66 (11.6)
Other/unknown	78 (13.7)
Disease severity	*n* (%)
Hospitalization	34 (6.0)
Oxygen therapy	12 (2.1)
Intensive care	2 (0.4)
Mechanical ventilation	1 (0.2)

^a^ Percentage among symptomatic individuals.

### 4.3 Longitudinal follow‐up

We detected five cases of probable reinfection and one confirmed reinfection between August 17, 2020, and March 1, 2022; their characteristics are reported in Table 4. We did not detect any possible reinfection. One participant reported a recurrence of acute symptoms 6 months after primary illness, but never tested positive again; this episode was excluded from analysis as it did not meet any primary outcome definition. Cumulative time at risk for probable reinfection amounted to 54 581 person‐days, for an average reinfection incidence rate of 4.0 (1.5–8.7) per 100 person‐year. Four reinfections (66%) were asymptomatic, whereas only 5% of initial infections were asymptomatic (Fisher exact test *p*‐value = 0.0001). No reinfections required hospitalization, whereas 6.0% of primary infections did (Fisher exact test *p*‐value = 1.00). One asymptomatic reinfection was detected through screening at enrollment, two were detected in the workplace, and another was detected through contact tracing. Viral loads in reinfection positive PCRs were generally low (see Table 4, cycle thresholds). Significant exposure within 2 weeks before the second positive PCR was reported in two (33%) reinfections.

**TABLE 4:** Characteristics of reinfection cases

Patient	Individual data	Comorbidity	Case definition	Δt D0 to 2nd PCR+	Δt 1st PCR+ to 2nd PCR+	Ct 2nd PCR+	1st infection	2nd infection	Last serology before 2nd PCR+	Significant exposure within 14 days of 2nd PCR+
1	Sex: F Age: 53 BMI: Normal Work: Nurse	None	Probable reinfection	50 days	156 days	N/A	Symptomatic Not hospitalized	Symptomatic Not hospitalized	Positive	Yes
2	Sex: F Age: 59 BMI: Overweight Work: Physician	None	Probable reinfection	7 days	168 days	34	Symptomatic Hospitalized (less than 1 day)	Asymptomatic	Positive	No
3	Sex: M Age: 43 BMI: Normal Work: Nurse	None	Probable reinfection	71 days	94 days	34	Symptomatic Hospitalized (less than 1 day)	Symptomatic Not hospitalized	Positive	No
4	Sex: M Age: 60 BMI: Overweight Work: Physician	Hypertension	Probable reinfection	0 days (detected at initial screening)	257 days	18	Symptomatic Not hospitalized	Asymptomatic	Positive	No
5	Sex: F Age: 32 BMI: Normal Work: Nurse	None	Probable reinfection	37 days	103 days	33	Symptomatic Not hospitalized	Asymptomatic	Negative	Yes
6	Sex: F Age: 32 BMI: Normal Work: Other	None	Probable reinfection	325 days	400 days	31	Asymptomatic	Asymptomatic	Negative	No

*Notes*: D0 = enrollment. PCR+ = positive PCR test. Δt = time interval. Ct = cycle threshold. First positive PCR refers to primary infection; second positive PCR refers to reinfection.

**FIGURE 2:**
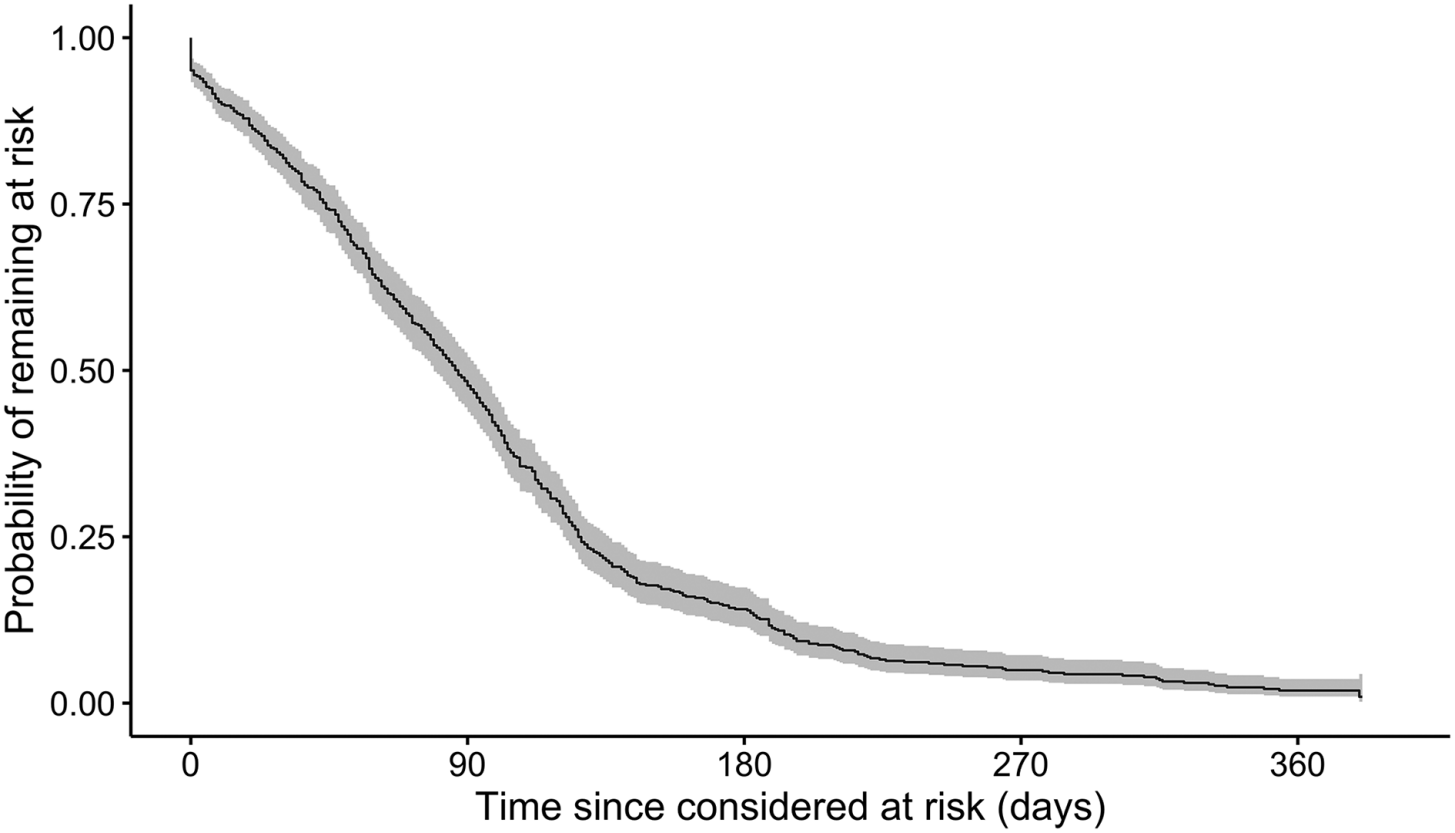
Kaplan–Meier curve for the probability of remaining in the pool of participants at risk of reinfection while unvaccinated, as a function of time since enrollment. The event of interest was reinfection or vaccination

We performed time‐to‐event Kaplan–Meier analysis for the probability of remaining at risk of primary outcome as function of time since enrollment (Figure 2). The median time at risk of primary outcome was 87 days (95% CI: 79–94). Removal from the at‐risk pool was primarily due to vaccination rather than reinfection. Most participants (533) received at least one dose of vaccine against SARS‐CoV‐2. Median time between enrollment and reception of first dose was 85 days (IQR: 51–124 days).

Seventy significant exposures to infectious COVID‐19 cases, documented through biweekly questionnaires, occurred in 40 distinct HCWs during their at‐risk period, for an incidence rate of 47 (36–59) significant exposures per 100 person‐years (see Supporting Information for more details).

**FIGURE 3:**
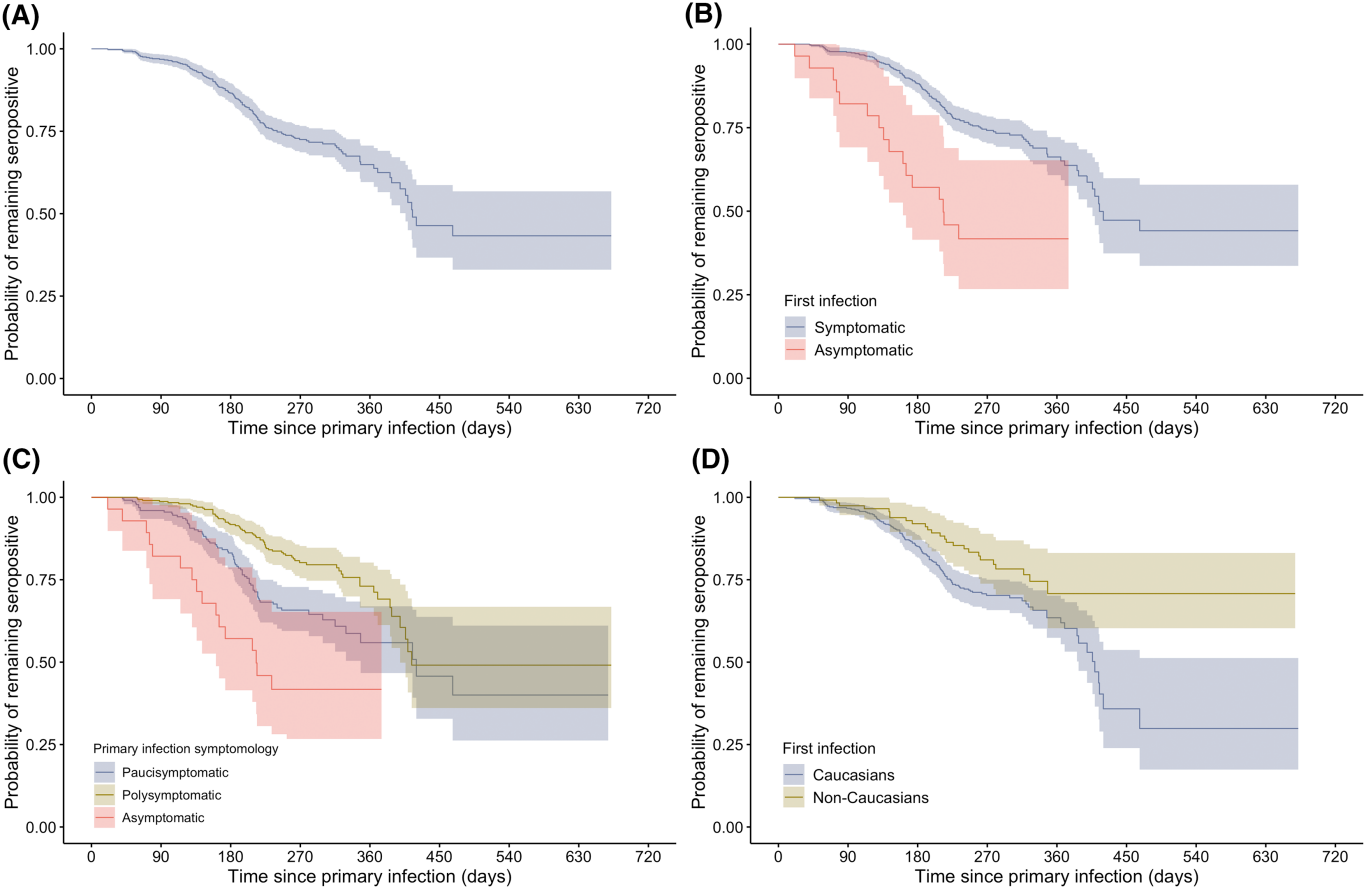
Kaplan–Meier curves for the probability of remaining seropositive as function of time since primary infection, in absence of vaccination. Negative serology was the primary event and vaccination was a censoring event. Panel A: Crude Kaplan–Meier curve including all participants. Panel B: Kaplan–Meier curves stratified by symptomology of primary infection (asymptomatic and symptomatic categories). Panel C: Kaplan–Meier curves stratified by symptomology of primary infection (asymptomatic, paucisymptomatic, and polysymptomatics categories). Panel D: Kaplan–Meier curves stratified by ethnicity (Caucasian and non‐Caucasian categories)

We performed time‐to‐event Kaplan–Meier analysis on our serology data, where the event was a negative serology test. The crude probability of remaining seropositive as a function of time since primary infection and in the absence of vaccination is shown in Figure 3, Panel A. One hundred sixty‐three (160) participants had a negative serology either at D0 (*n* = 138) or during follow‐up (*n* = 22). The crude median duration of seropositivity was 415 days since primary infection (95% CI: 406–infinity).^‡^

We also produced Kaplan–Meier curves stratified by presence/absence of symptoms at primary infection (Figure 3, Panel B), by number of symptoms at primary infection (Figure 3, Panel C), and by ethnicity (Figure 3, Panel D). For this analysis, paucisymptomatic primary infection was defined as a primary infection with one to seven distinct symptoms and polysymptomatic primary infection was defined as a primary infection with eight or more distinct symptoms. The median duration of seropositivity was 213 days (95% CI: 161–infinity) in participants with asymptomatic primary infection compared with 415 days (95% CI: 406–infinity) in participants with symptomatic primary infection. Stratification by number of symptoms yielded a median duration of seropositivity of 420 days (95% CI: 348–infinity) in participants with paucisymptomatic primary infection compared with 414 days (95% CI: 399–infinity) in participants with polysymptomatic primary infection. Median duration of seropositivity in non‐Caucasians was undefined,^§^ whereas median duration of seropositivity in Caucasians was 409 days (95% CI: 388–infinity).

We performed multivariate Cox regression on our serology data to adjust for potential confounders; the event of interest was again a negative serology. Participants with asymptomatic primary infection were less likely to remain seropositive over time [adjusted HR = 2.19 (1.26–3.81)] when compared with participants with paucisymptomatic primary infection (reference category). Participants with polysymptomatic primary infection were more likely to remain seropositive during the first 300 days since primary infection [adjusted HR = 0.48 (0.34–0.70)] when compared with participants with paucisymptomatic primary infection. After 300 days since primary infection, no significant difference between polysymptomatics and paucisymptomatics was observed [adjusted HR = 1.35 (0.51–3.58)]. Participants with obesity [adjusted HR = 0.51 (0.32–0.84)], age over 55 years [adjusted HR = 0.52 (0.29–0.91)], and non‐Caucasian ethnicity [adjusted HR = 0.51 (0.33–0.79)] were more likely to remain seropositive over time (see Supporting Information for details).

Sixty‐four participants (11.2%) were either lost to follow‐up or withdrew before end of study.

## 5 DISCUSSION

We described the results of the RECOVER study over 18 months of follow‐up. Our study shows that reinfection in unvaccinated HCWs with a history of PCR‐confirmed SARS‐CoV‐2 infection remains a rare event over the first year after primary infection. Our measured reinfection incidence rate of 4.0 per 100 person‐year is generally concordant with rates observed by other authors in HCWs and the general population. By comparison, Gallais et al observed a reinfection incidence rate of 0.40 per 100 person‐years in a cohort of French HCWs over 13 months.^19^ Hall et al followed a cohort of English HCWs prospectively for 1 year (SIREN study) and observed a reinfection incidence rate of 2.8 per 100 person‐year.^7^ Another English cohort study in HCWs by Lumley et al reported a reinfection incidence rate of 0.47 per 100 person‐years.^9^ Other studies investigated reinfection rates in the general population. Abu‐Raddad et al estimated the reinfection incidence rate in Qatar at 1.3 per 100 person‐years using a cohort of laboratory‐confirmed primary infections.^20^ In a Danish population‐level observational study by Hansen et al, the reinfection incidence rate was estimated at 2.0 per 100 person‐years.^12^ Other studies reported risk of reinfection in various cohorts,^6^, ^21^, ^22^, ^23^ but did not report incidence rates.¶ It is important to note that the reinfection rates observed in these studies predate the Omicron wave and depend on the incidence rates in the general population of each region/country, which limits inter‐study comparability.

Our measured incidence rate of self‐reported significant exposure was approximately 12 times higher than our reinfection incidence rate. However, it remains unclear if this rate ratio constitutes a reliable measure of protection because (i) we did not have a cohort naive to SARS‐CoV‐2 to establish baseline comparison values for infection and significant exposure incidence rates and (ii) it is likely that many significant exposure events were either unreported or unrecognized by participants.

Our Kaplan–Meier analysis shows that the overall probability of remaining seropositive up to 300 days after the initial infection is approximately 70% in the absence of vaccination. Persistence of seropositivity was positively correlated with the number of symptoms at primary infection for the first 300 days after primary infection. Beyond 300 days postinfection, we did not find a significant impact of symptomology on the hazard rate of seronegative tests. This suggests that the impact of symptomology at primary infection on seropositivity is relatively short‐lived. Median duration of seropositivity was significantly longer in non‐Caucasian participants compared with Caucasian participants. Although not explained in our regression models, we hypothesize that the effect of ethnicity on duration of seropositivity could be attributed to profession, workplace, and household size,^24^, ^25^ because these characteristics could provide differential levels of undetected re‐exposures between ethnicity categories. Finally, it remains uncertain whether positive serology constitutes a strong correlate of protection.^26^ However, emerging evidence indicates that higher levels of IgG seem correlated with higher neutralization capacities.^7^, ^12^, ^26^

Our epidemiological and serological evidence supports the hypothesis that primary infection by SARS‐CoV‐2 confers significant protection against reinfection for at least several months.

Our study has several strengths. First, our prospective cohort is representative of the population of HCWs in the Greater Montreal area through our multicentric recruitment process and permissive eligibility criteria. Participants were closely monitored during follow‐up, which decreases recall/memory bias. Data collection was exhaustive, allowing adjustment/stratification for many potential confounders. HCWs are probably more frequently exposed to SARS‐CoV‐2 than the general population, increasing the validity of our measurements. Finally, nearly 90% of participants remained enrolled for the full duration of the study, limiting selection bias from loss to follow‐up.

Our study nevertheless has some limitations. First, we could not enroll a cohort of SARS‐CoV‐2‐naive HCWs for comparison. Therefore, we could not provide a numerical estimate of the protection conferred by natural infection. We observed a rather small number of reinfections, hence limiting statistical power. We could not determine whether specific individual characteristics were associated with an increased or decreased reinfection probability. It is also likely that we missed cases of asymptomatic reinfections, because we did not systematically screen all participants with NP swabs on a regular basis. Our measured reinfection incidence rate thus probably underestimates the true reinfection rate. Our study could not identify any case of confirmed reinfection by full‐genome sequencing, because material recovered in reinfection swabs was insufficient. These low viral loads suggest that reinfected individuals may be less likely to transmit SARS‐CoV‐2 to susceptible individuals compared with naive individuals who become infected.^27^ In addition, most participants were already vaccinated when the Omicron variant started spreading significantly. This limits our study's power to investigate the difference between Omicron's reinfection potential and the Alpha and Delta variants.

Vaccines against SARS‐CoV‐2 became available to participants about 4 months after the initiation of our study. Time between enrollment and vaccination was highly heterogeneous, which could have imparted selection bias; it also precluded the calculation of a meaningful risk of reinfection while unvaccinated over 12 months.

Finally, our cohort is composed mostly of young, female, Caucasian and generally healthy HCWs. This limits the generalizability of our results to other populations.

## 6 SUMMARY

Reinfection by SARS‐CoV‐2 remained a rare event among a population of 569 Canadian HCWs over 12 months of follow‐up. Reinfection episodes were milder than original illness and were characterized by low viral loads. Primary infection induced detectable serum IgG levels in the majority (75%) of participants at enrollment. Duration of seropositivity was positively correlated to the following individual characteristics: age 55 years and above, obesity, and non‐Caucasian ethnicity. Number of symptoms at primary infection was also positively correlated with seropositivity over the first 300 days postinfection. Our study provides epidemiological and serological evidence that initial infection by SARS‐CoV‐2 confers protection against reinfection for several months. Additional research is needed to assess the frequency of asymptomatic reinfections and their relative transmissibility compared with primary infections. There is also a need to complement these findings with an in‐depth analysis of the humoral and cellular responses to SARS‐CoV‐2 infection.

## AUTHOR CONTRIBUTIONS

**Étienne Racine:** Data curation; formal analysis; methodology; software; validation; visualization. **Guy Boivin:** Conceptualization; funding acquisition; investigation; methodology; resources; supervision; validation. **Yves Longtin:** Conceptualization; funding acquisition; methodology; resources. **Deirdre McCormack:** Conceptualization; investigation; project administration; resources. **Hélène Decaluwe:** Conceptualization; formal analysis; funding acquisition; investigation; methodology; resources; supervision; validation. **Patrice Savard:** Funding acquisition; resources; validation; visualization. **Matthew Cheng:** Funding acquisition; methodology; resources; validation. **Marie‐Ève Hamelin:** Data curation; investigation. **Julie Carbonneau:** Investigation. **Fazia Tadount:** Data curation; methodology; project administration. **Kelsey Adams:** Data curation; formal analysis; investigation; project administration; resources; software; validation; visualization. **Benoîte Bourdin:** Visualization. **Sabryna Nantel:** Investigation; software. **Vladimir Gilca:** Funding acquisition. **Jacques Corbeil:** Conceptualization; funding acquisition; methodology. **Gaston De Serres:** Conceptualization; formal analysis; funding acquisition; methodology; validation.

## CONFLICT OF INTEREST

All authors state that they have no financial relationships or conflicts of interest relevant to this article to disclose.

### PEER REVIEW

The peer review history for this article is available at https://publons.com/publon/10.1111/irv.12997.

## Supporting information


**Table S1**: Multivariate logistic regression model for the odds of hospitalization as function of known severe COVID‐19 risk factors.
**Table S2**: Demographic characteristics of the RECOVER participants with at least 1 significant exposure event while at risk of probable reinfection.
**Table S3**: Multivariate Cox regression model for the hazard of testing seronegative. Period 1: less than 300 days since primary infection. Period 2: 300 days and over since primary infection.
**Figure S1**: Kaplan–Meier curve for the probability of reporting symptoms attributable to the primary infection, as a function of time since primary infection. Participants with asymptomatic primary infection were excluded from this analysis.

## Data Availability

Data are currently not publicly available but may be shared upon request with research protocol.

## ENDNOTES

* Number limited to 30 because of budget constraints.

^†^
As reported by participants.

^‡^
Assuming all participants were seropositive at time of primary infection.

^§^
Because less than 50% of non‐Caucasians became seronegative during follow‐up, prior to vaccination.

¶ Peer‐reviewed reinfection incidence rates cited in this section were estimated before the spread of the Omicron variant; they may not reflect reinfection incidence rates during the first Omicron wave.
