# Supplementary material for: The REinfection in COVID‐19 Estimation of Risk (RECOVER) study: Reinfection and serology dynamics in a cohort of Canadian healthcare workers
Source: Influenza Other Respir Viruses. 2022 May 5;16(5):916–25. doi: 10.1111/irv.12997 (PMC9343327; doi:10.1111/irv.12997)
Supplement: Supplementary file 1 — Table S1: Multivariate logistic regression model for the odds of hospitalization as function of known severe COVID‐19 risk factors. Table S2: Demographic characteristics of the RECOVER participants with at least 1 significant exposure event while at risk of probable reinfection. Table S3: Multivariate Cox regression model for the hazard of testing seronegative. Period 1: less than 300 days since primary infection. Period 2: 300 days and over since primary infection. Figure S1: Kaplan–Meier curve for the probability of reporting symptoms attributable to the primary infection, as a function of time since primary infection. Participants with asymptomatic primary infection were excluded from this analysis. [file IRV-16-916-s001.docx]

The REinfection in COVID-19 Estimation of Risk (RECOVER) study: Reinfection and serology dynamics in a cohort of Canadian healthcare workers.

SUPPLEMENTARY MATERIAL

METHODS

*Study timeline*

Participants were recruited between August 17^th^, 2020, and April 8^th^, 2021. Recruited participants acquired their primary infection during the first wave (Spring 2020) or the second wave of the pandemic (Fall 2020 – Winter 2021). Primary infections were acquired before variants of concern were introduced in Canada. The follow-up period for results presented in this article begins August 17^th^, 2020 and ends March 1^st^, 2022, during which all variants of concern circulated significantly, including Omicron.

*Cohort size*

Sample size was designed to measure a 3% risk of reinfection over 12 months with 95% confidence of a ±1.5% precision (half the estimated risk). During study design, estimates of reinfection risk were obtained from literature on other human coronaviruses, which suggested a higher risk of reinfection than what has since been observed with SARS-CoV-2, except possibly with the recent Omicron variant. Vaccines were in development and were not available at the time of study design. Accounting for an estimated 10 to 15% loss to follow-up, a sample size of 570 HCWs was deemed optimal [1].

*Data collection, management, and analysis*

Demographic data included date of birth, biological sex at birth, ethnicity, workplace, and profession. Clinical data included height, weight, medical history, medication, lifestyle information, and recent vaccination history. Detailed information about original COVID-19 illness was collected, including symptomatology, duration of symptoms, date of first positive PCR test, and need for hospitalization/advanced treatment.

Data was extracted from REDCap and exported into CSV/Microsoft Excel worksheets for initial management and then imported into RStudio for all analyses. Tolerance threshold to type I error was defined as $\alpha$ = 0.05. Log-rank *p*-values derived from the score test were used for statistical comparison of Kaplan-Meier curves. Cox regression modeled the hazard to obtain a negative serology test, i.e. the probability per unit time to obtain a seronegative test conditional to being seropositive at the previous test. Therefore, a hazard ratio (HR) higher than 1 represents a shorter average duration of seropositivity.

RESULTS

*Persistence of symptoms*

Figure S1 shows the probability of remaining symptomatic as function of time since primary infection. For participants who reported no residual symptoms at enrolment (D0), the duration of (acute) symptoms was collected by questionnaire at D0. Participants who reported residual symptoms at D0 were considered symptomatic until they declared complete resolution of symptoms at a quarterly visit. Recurrent symptoms, defined as symptoms reappearing after resolution, were not considered in this article. More than half of participants reported residual symptoms 3 months after primary infection; about 30% of participants reported residual symptoms one year after primary infection. The median time to resolution of symptoms was 248 days (95% CI: 217 – 294).

*Logistic regression model for hospitalization risk factors*

We investigated whether known risk factors for severe COVID-19 were significant predictors of hospitalization risk in our cohort. We used odds ratios derived from penalized maximum likelihood (PLM) logistic regression (R package *logistf* ) to estimate the association between potential risk factors and probability of hospitalization. We chose this approach since the outcome is binary, has relatively low prevalence (n=34, 6.0%) and cell counts in some categories are low. Regression results for the base model are shown in Table S1.

Model results did not vary significantly when changing the age cutoff defining the older age category. The baseline model with cutoff at 55 years of age had the lowest AIC value and has thus been retained as the best model. Obesity was the only covariate significantly associated with higher odds of hospitalization.

*Significant exposures*

Forty HCWs reported significant exposures to SARS-CoV-2 during longitudinal follow-up. The demographic characteristics of this subgroup are reported in Table S2.

*Cox regression model for serology time series data*

We built an exploratory Cox regression model to investigate which individual factors, if any, act as predictors of persistence of IgG seropositivity after primary infection. The event of interest for this survival model was a negative serology test. Regression models were built sequentially with the *coxph*() function to identify candidate predictors and potential confounders. To satisfy the proportional hazards assumption, the Cox model was split over two time periods (< 300 days since primary infection and ≥ 300 days since primary infection) for two covariates: male sex and polysymptomatic primary infection. The split in two time periods for sex and polysmptomatic covariates was performed with the *survSplit*() function.

Coefficients of the final model and their confidence intervals are shown in Table S3. The proportional hazard hypothesis was tested with the *cox.zph*() function. The global *p*-value of the proportional hazards hypothesis test was 0.147. The *p*-values of the proportional hazards hypothesis test for each model coefficient were all less than 0.05.

Further adjustment for smoking/vaping, vitamin D intake, workplace, profession, and household size did not significantly change model results. These variables were not retained for the final model for sake of simplicity.

REFERENCE

1. Naing L, Winn T, Nordin R. Pratical Issues in Calculating the Sample Size for Prevalence Studies. Arch Orofac Sci **2006**; 1.

**Table S1** : Multivariate logistic regression model for the odds of hospitalization as function of known severe COVID-19 risk factors.

| Characteristic | Odds ratio of hospitalization | 95% CI | *p*-value |
| --- | --- | --- | --- |
| Male sex | 1.41 | 0.59 – 3.13 | 0.429 |
| Age over 55 years | 2.39 | 0.98 – 5.45 | 0.054 |
| Non-Caucasian ethnicity | 1.26 | 0.52 – 2.77 | 0.592 |
| Obese | 2.80 | 1.15 – 7.02 | 0.023 |
| Overweight | 1.25 | 0.49 – 3.22 | 0.636 |
| Underweight | 1.61 | 0.012 – 15.1 | 0.767 |
| Comorbidity | 1.17 | 0.44 – 2.86 | 0.747 |
| Smoking/vaping | 0.16 | 0.001 – 1.2 | 0.088 |

**Table S2**: Demographic characteristics of the RECOVER participants with at least 1 significant exposure event while at risk of probable reinfection.

| Sex at birth | n (%) |
| --- | --- |
| Female | 33 (82.5) |
| Male | 7 (17.5) |
| Age | Years |
| Median (IQR) | 33 (13.5) |
| Range | 21 – 59 |
| Ethnicity | n (%) |
| Caucasian | 32 (80.0) |
| Other | 8 (20.0) |
| Workplace | n (%) |
| Hospital | 20 (50.0) |
| Public long-term care facility | 7 (17.5) |
| Community health center | 5 (12.5) |
| Private care facility | 1 (2.5) |
| Other | 7 (17.5) |
| Staff group | n (%) |
| Medical doctor/resident | 6 (15.0) |
| Nurse/paramedic | 18 (45.0) |
| Patient care attendant | 10 (25.0) |
| Therapist/other healthcare professional in regular contact with individual patients | 3 (7.5) |
| Other | 3 (7.5) |

**Table S3**: Multivariate Cox regression model for the hazard of testing seronegative. Period 1: less than 300 days since primary infection. Period 2: 300 days and over since primary infection.

| Characteristic | Hazard ratio | 95% CI | *p*-value |
| --- | --- | --- | --- |
| Asymptomatic primary infection | 2.19 | 1.26 – 3.81 | 0.006 |
| Polysymptomatic primary infection (period 1) | 0.48 | 0.34 – 0.70 | 0.0001 |
| Polysymptomatic primary infection (period 2) | 1.35 | 0.51 – 3.58 | 0.548 |
| Hospitalization | 0.39 | 0.12 – 1.22 | 0.105 |
| Male sex (period 1) | 0.94 | 0.58 – 1.51 | 0.795 |
| Male sex (period 2) | 2.11 | 0.59 – 7.46 | 0.248 |
| Age over 55 years | 0.50 | 0.28 – 0.89 | 0.019 |
| Any comorbidity | 1.22 | 0.71 – 2.11 | 0.469 |
| Non-Caucasian | 0.51 | 0.33 – 0.80 | 0.003 |
| Obese | 0.52 | 0.32 – 0.85 | 0.009 |
| Overweight | 0.96 | 0.67 – 1.37 | 0.807 |
| Underweight | 1.53 | 0.47 – 4.94 | 0.477 |

**Figure S1**: Kaplan-Meier curve for the probability of reporting symptoms attributable to the primary infection, as a function of time since primary infection. Participants with asymptomatic primary infection were excluded from this analysis.
